# ZmbZIP60 mRNA is spliced in maize in response to ER stress

**DOI:** 10.1186/1756-0500-5-144

**Published:** 2012-03-14

**Authors:** Yanjie Li, Sabrina Humbert, Stephen H Howell

**Affiliations:** 1 Plant Sciences Institute, Iowa State University, Ames IA 50014, USA; 2 Molecular and Cellular Biology Department, University of Guelph, Guelph Ontario N1G2W1, Canada

**Keywords:** IRE1, bZIP transcription factor, mRNA splicing, Unfolded protein response, Binding protein, Chaperone, Heat stress, Corn

## Abstract

**Background:**

Adverse environmental conditions produce ER stress and elicit the unfolded protein response (UPR) in plants. Plants are reported to have two "arms" of the ER stress signaling pathway-one arm involving membrane-bound transcription factors and the other involving a membrane-associated RNA splicing factor, IRE1. IRE1 in yeast to mammals recognizes a conserved twin loop structure in the target RNA.

**Results:**

A segment of the mRNA encoding ZmbZIP60 in maize can be folded into a twin loop structure, and in response to ER stress this mRNA is spliced, excising a 20b intron. Splicing converts the predicted protein from a membrane-associated transcription factor to one that is targeted to the nucleus. Splicing of ZmbZIP60 can be elicited in maize seedlings by ER stress agents such as dithiothreitol (DTT) or tunicamycin (TM) or by heat treatment. Younger, rather than older seedlings display a more robust splicing response as do younger parts of leaf, along a developmental gradient in a leaf. The molecular signature of an ER stress response in plants includes the upregulation of Binding Protein (BIP) genes. Maize has numerous BIP-like genes, and ER stress was found to upregulate one of these, ZmBIPb.

**Conclusions:**

The splicing of ZmbZIP60 mRNA is an indicator of ER stress in maize seedlings resulting from adverse environmental conditions such as heat stress. ZmbZIP60 mRNA splicing in maize leads predictively to the formation of active bZIP transcription factor targeted to the nucleus to upregulate stress response genes. Among the genes upregulated by ER stress in maize is one of 22 BIP-like genes, ZmBIPb.

## Background

Crop production worldwide suffers major losses each year from abiotic stresses such as drought, flooding, heat and saline conditions [1]. It is estimated (from the empirical yield gap analysis) that more than 50% of the potential yield in maize is lost to abiotic stress [2], making stress tolerance a critical trait for crop improvement. In fact, it has been argued that much of the genetic yield gain achieved in the past 40 years in maize is due to greater stress tolerance rather than to an increase in yield potential [3-7].

A sentinel for adverse environmental conditions in plants is the unfolded protein response (UPR) [8]. Considered an environmental stress sensor in plants, UPR is set off by the accumulation of unfolded or misfolded proteins in the ER. Protein folding is a finicky process characterized by an energy landscape with many peaks and valleys, and under adverse environmental conditions protein folding can easily be disrupted [9]. UPR in plants is characterized by the upregulation of genes encoding chaperones and components of protein folding or protein degradation systems [10]. The products of these genes mitigate the damage caused by ER stress and help to reduce the load of misfolded proteins in stressed cells.

UPR was first described in plants a number of years ago [11-13]-recognized by the upregulation of genes characteristic of the response [14]. However, it has only been in the past few years that the components of the ER stress signaling pathway were identified in plants [8]. So far, two "arms" of the ER stress signaling pathway have been described in plants. One arm involves the proteolytic cleavage of ER membrane-associated transcription factors (TFs). In Arabidopsis, two of these membrane-associated TFs have been identified, bZIP17 [15] and bZIP28 [16-18]. In response to ER stress, these TFs are mobilized from the ER and move to the Golgi, where they are processed, and their TF components are released by Golgi-resident proteases [18,19]. The TFs relocate to the nucleus where they upregulate stress response genes [20].

The other arm of the ER stress pathway involves a non-conventional RNA splicing event meditated by an evolutionarily conserved ER stress sensor, IRE1. IRE1 is an ER membrane localized stress transducer with protein kinase and ribonuclease activities [21,22]. In yeast, IRE1 is thought to be directly activated by misfolded proteins in the ER [23], and when activated, IRE1 dimerizes or oligomerizes [24] and splices a target mRNA-Hac1. In mammalian cells, XBP1 mRNA has been identified as an IRE1 target. In both cases, the splicing event leads to the production of active TFs, which, in turn, upregulate the expression of ER stress response genes.

Although homologs of yeast and mammalian IRE1s were reported some time ago in Arabidopsis, IRE1a and b [25,26], their mRNA targets were not known. Only recently did the identity of the target mRNA come to light when a mRNA coding AtbZIP60 was found containing an IRE1 recognition site [27]. The recognition site consists of two kissing hairpin loops each of which contains 3 conserved bases [28,29]. It was subsequently demonstrated that AtbZIP60 mRNA was spliced in response to treatment of seedlings with ER stress agents [27]. The unspliced form of bZIP60 mRNA encodes a bZIP TF with a transmembrane domain (TMD). Splicing creates a frame shift downstream of the bZIP domain such that the spliced form encodes a TF lacking a TMD but having acquired a nuclear localization signal [27,30]. This form is located in nucleus where it upregulates stress response genes.

The question we address in this study is whether maize has the same RNA splicing arm of the ER stress response pathway, and, if so, what are the differences in this response in monocots compared to dicots.

## Results

### ZmbZIP60 mRNA splicing

AtbZIP60 is a member of a subfamily of bZIP transcription factors in Arabidopsis, which have transmembrane domains and are predicted to be membrane-associated transcription factors. An ortholog of AtbZIP60 was identified in maize (GB: AY104864) by blast search. The predicted amino acid sequence of the protein encoded by the maize gene (hereafter called ZmbZIP60) was ~29% identical to that of Arabidopsis. In general, the bZIP60-related genes in other monocots and dicots appear to constitute separate clades (Additional file 1).

In response to ER stress, AtbZIP60 is spliced by an ER-localized ribonuclease, IRE1 [27]. There are two IRE1 genes in Arabidopsis, *IRE1a *and *IRE1b *[26], and *IRE1b *is responsible for most of the bZIP60 mRNA splicing in stressed seedlings [27]. Maize also has two IRE1 orthologs, although it is not clear which one is orthologous to AtIRE1a and which to AtIRE1b, since IRE1-like genes in dicots and monocots lie in two separate clades with soybean IRE1 as an outlier (Additional file 2).

**Figure 1:**
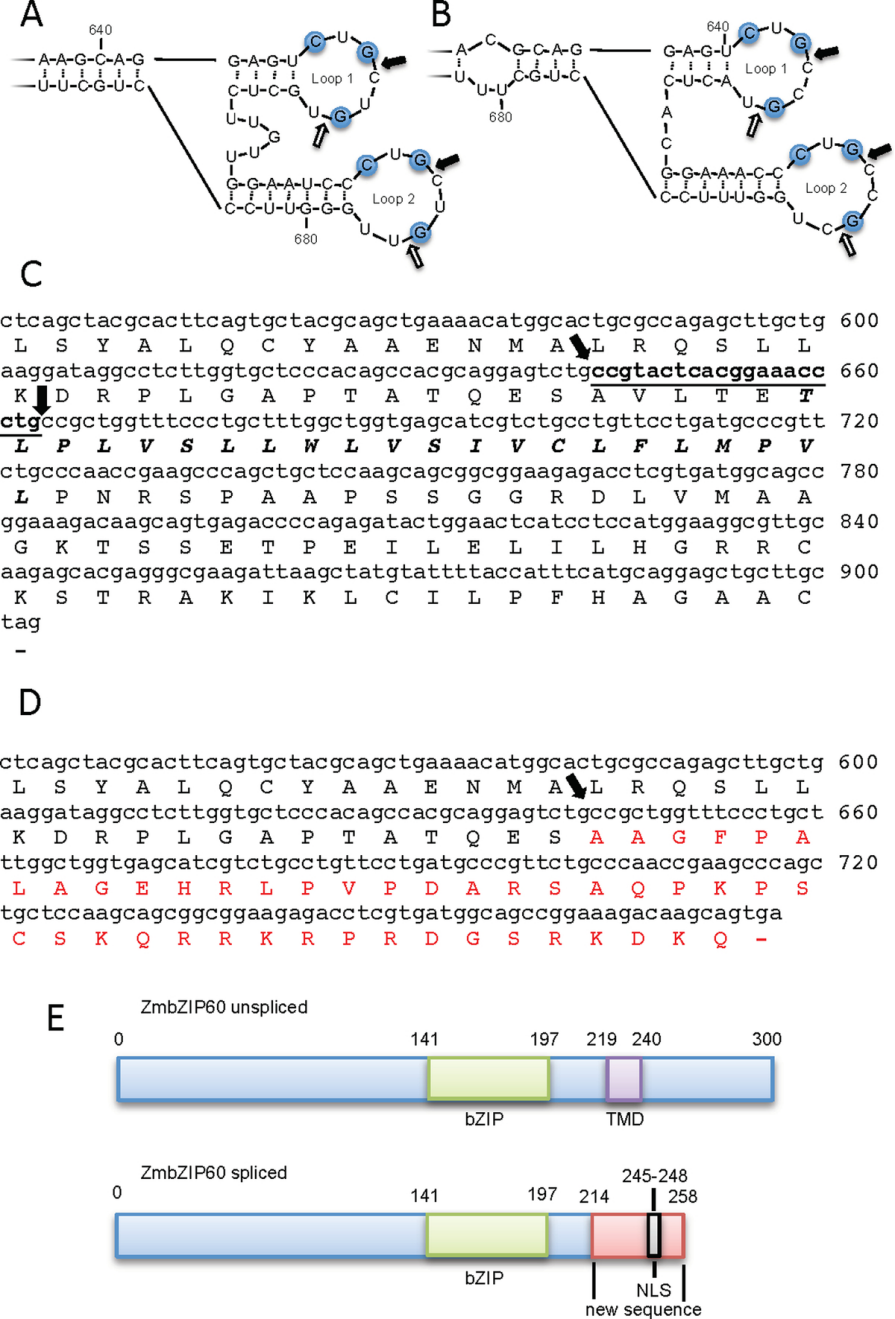
**IRE1 recognition sites in ZmbZIP60 mRNA and predicted protein**. Diagram comparing RNA secondary structure predicted from Mfold for a segment of (A) AtbZIP60 mRNA and (B) ZmbZIP60 mRNA. (C) Partial predicted protein sequence encoded by unspliced ZmbZIP60 mRNA. Splice sites are indicated by arrows and the 20b intron is underlined. The predicted TMD is indicated in italics. (D) Partial predicted protein sequence encoded by spliced ZmbZIP60 mRNA. The arrow indicates the splice junction. The novel sequence produced by the frameshift is indicated in red. The putative nuclear localization signal is underlined. (E) Diagram showing the structures of the predicted proteins encoded by unspliced and spliced ZmbZIP60 mRNAs.

The consensus substrate recognition sequence for IRE1 is a pair of kissing hairpin loops with three conserved bases in each loop [28]. Both maize and Arabidopsis bZIP60 mRNAs fold into comparable structures (Figure 1A and 1B). All of the bZIP60-related plant genes identified in this study by blast searches have sequences predicted to form IRE1 recognition sites (Additional file 3). In general, the sites in monocots appear to have a shorter interloop bridge sequence than dicots.

**Figure 2:**
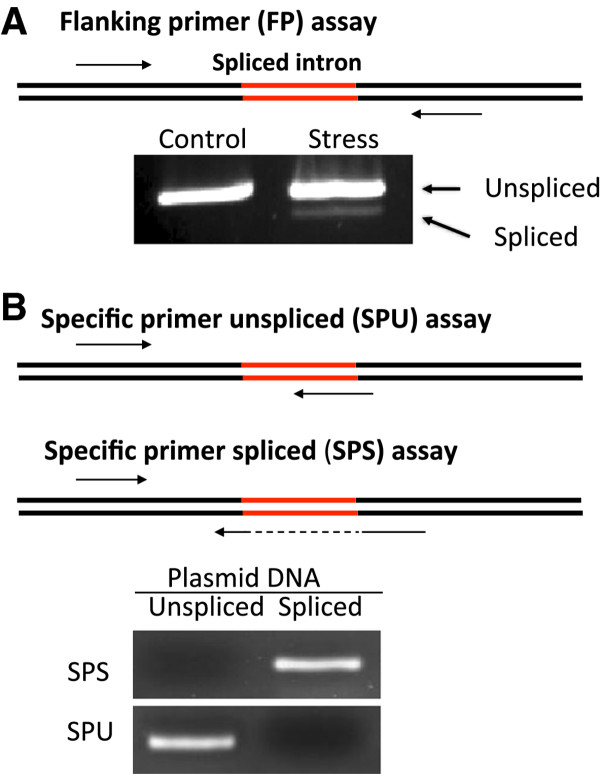
**PCR assays used in this study**. (A) Flanking primer (FP) assay employs primers immediately flanking the splice site, so PCR products from both the unspliced and spliced Zmbzip60 are amplified. (B) Specific primer unspliced (SPU) assay uses a primer that crosses the exon-intron boundary and is specific for detecting the unspliced form of bzip60. Specific primers spliced (SPS) assay uses a primer that crosses the exon-exon boundary and is specific for detecting the spliced form of ZmbZIP60. The specificity of the two SP assays was demonstrated by amplifying the plasmid-cloned cDNA derived from the spliced and unspliced bzip60.

To determine if ZmbZIP60 is spliced in a manner similar to AtbZIP60, we exposed corn seedlings to ER stress agents such as dithiothreitol (DTT) and tested for ZmbZIP60 splicing using RT-PCR assays. Two assays were used as described before [27]-one, a flanking primers (FP) assay, which detects both the unspliced and spliced bZIP60 mRNAs (Figure 2A), and another, a specific primers (SP) assay, which detects either the spliced (SPS) or the unspliced (SPU) forms of bZIP60 (Figure 2B).

Exposure of corn seedlings to DTT, did, indeed, result in splicing of ZmbZIP60, and the spliced form was recovered and sequenced. The sequence revealed that a 20b segment had been excised from the mRNA (Figure 1C and 1D) brought about by cleavages in the twin loops as indicated (Figure 1B). The unspliced mRNA is predicted to encode a type II membrane protein of 300 AA with a single transmembrane domain (TMD) (Figure 1C and 1E). The excision of a 20b segment produces a frameshift such that the spliced form is predicted to encode a protein of 258 AA lacking a TMD, but having acquired a putative nuclear targeting signal (NLS) (Figure 1D and 1E). Thus, splicing of ZmbZIP60 mRNA converts a mRNA from one encoding a membrane-bound transcription factor to a soluble transcription factor that is targeted to the nucleus.

### Stress agents

**Figure 3:**
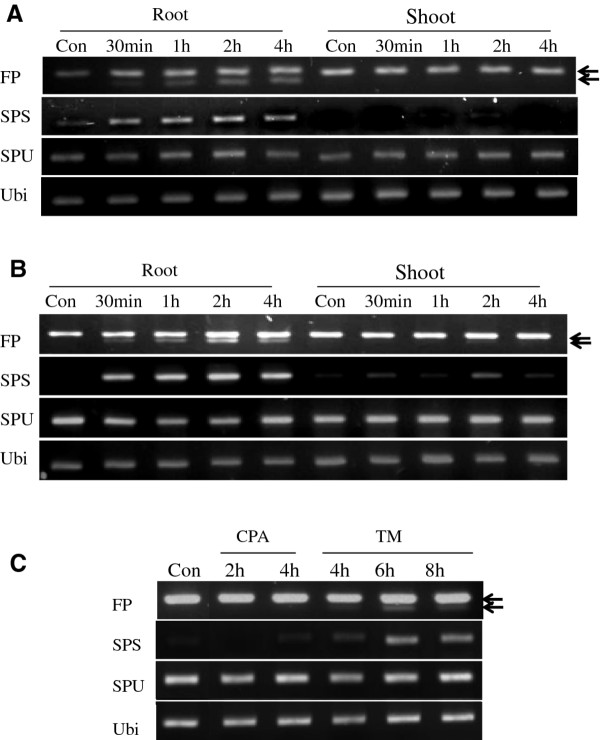
**Induction of ZmbZIP60 splicing by ER stress agents**. ZmbZIP60 splicing in maize seedlings was detected using a flanking primers RT-PCR assay (FP) or a specific primer assay for spliced mRNA (SPS) or unspliced mRNA (SPU). Both unspliced (slower migrating) and spliced (faster migrating) forms appear in the FP assay and the migration position of both forms is indicated by arrows. Seven-day-old seedlings were treated by (A) immersing their roots in 2.5 mM DTT solution or (B) topically spraying the seedlings with the same solution. RNA was extracted from roots and shoots. (C) Seedlings were treated with 100 μM cyclopiazonic acid (CPA) or with 5 μg/ml tunicamycin (TM). RNA was obtained from roots. Con = control (No treatment).

Having a mRNA splicing assay in hand, we are able to test various stress agents for their ability to elicit the splicing response in corn seedlings. Seeds were germinated on moist filter paper. Typically, stress agents such as DTT or tunicamycin are used in the laboratory to elicit ER stress responses. They serve as proxies for environmental stresses by interfering with protein folding in the ER. In our experiments, the agents were either added to a hydroponic solution or topically applied by spraying directly onto the seedlings. When DTT was added to the hydroponic medium, spliced forms of ZmbZIP60 were detected by the FP assay in root tissue 30 min after treatment and increased thereafter (Figure 3A). The SPS assay is more sensitive, and it can be clearly seen that spliced forms are induced by 30 min after DTT treatment. There does appear to be some increase in the unspliced form in roots following DTT treatment, but that effect was not reproducible. No splicing was observed in the shoots.

The failure to observe splicing in shoots could be due to the fact that DTT is not rapidly transported to shoots when administered in the hydroponic solution. However, RNA splicing was not observed in shoots when the seedlings were sprayed with a DTT-containing solution, even though the splicing response was observed in roots treated in the same manner (Figure 3B). Still, this experiment does not rule out the possibility that shoots might respond to DTT treatment as it is possible that the leaf cuticle may have prevented DTT from entering the cells. Two other stress agents were tested, cyclopiazonic acid (CPA) and tunicamycin (TM), and administered by spraying and sampling root tissue after treatment. CPA inhibits ER Ca(2+)-ATPase activity [31] and TM prevents N-glycosylation [32], both of which interfere with protein folding in the ER. CPA did not elicit ZmbZIP60 mRNA splicing, but TM did, although the effect was much slower than treatment with DTT (Figure 3C). Again, the failure to observe an effect by CPA and slowness of the TM response may be due to the slow uptake of these agents. Because of the rapid action of DTT, this chemical was used in subsequent experiments as a stress-inducing agent in corn.

### Environmental stresses

**Figure 4:**
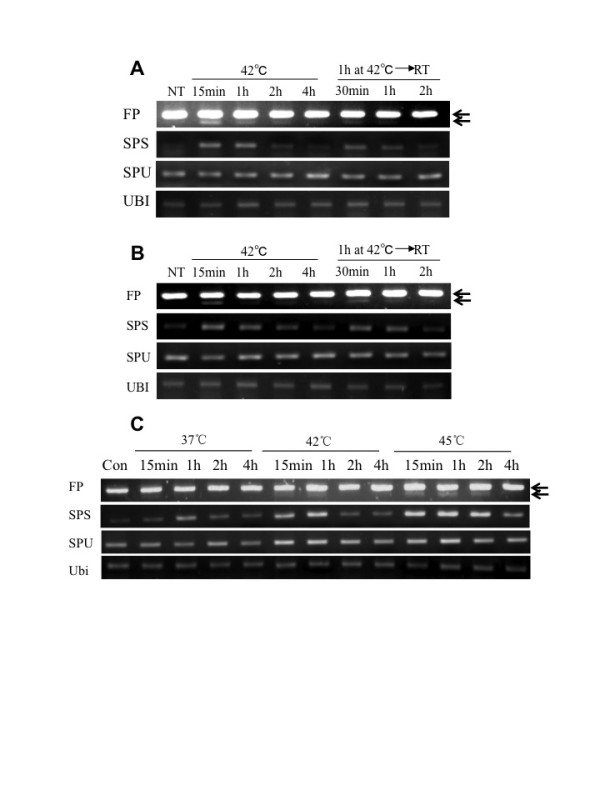
**Induction of ZmbZIP60 splicing under heat stress**. Maize seedlings were grown at room temperature (RT) and then incubated at 42°C for the times indicated. Some of the seedlings were recovered at RT after heat treatment for 1 hour. RNA was extracted from (A) roots and (B) shoots and analyzed using the RT-PCR assays described in Figure 2. (C) Temperature dependence of the RNA splicing reaction. Seedlings were incubated at elevated temperature for the times indicated.

It should be pointed out again that ER stress agents are conveniently used in the laboratory only as proxies to elicit ER stress. Therefore, we tested naturally occurring environmental stresses for their effects on ZmbZIP60 RNA splicing. In Arabidopsis, heat is an efficient inducer of UPR [27], and for this reason we treated corn seedlings with elevated temperatures. When seedlings were exposed to 42°C, ZmbZIP60 mRNA was rapidly spliced (within 15 min) in both roots (Figure 4A) and shoots (Figure 4B), while the level of unspliced RNA was about the same before and after treatment. Unlike treatment with ER stress agents, ZmbZIP60 was spliced in shoots in response to heat stress, demonstrating that shoots are capable of splicing ZmbZIP60 mRNA.

The splicing response was transient under constant heat (42°C) conditions (Figure 4A and 4B). The spliced form peaked at about 1 hr and was barely detectable by 4 hrs. Its signal rapidly diminished when seedlings were returned to room temperature after 1 hr exposure to 42°C. The amplitude of the splicing response appeared to be temperature dependent, at least up to 45°C (Figure 4C). As the amplitude increased, so did the length of the transient response period.

Other environmental stresses were tested for their ability to induce ZmbZIP60 mRNA splicing, such as hypoxia and cold (Additional file 4A) and NaCl, dehydration and abscisic acid (ABA) treatment (Additional file 4B and 4C). None of these elicited splicing under our conditions.

### Responses during plant development

**Figure 5:**
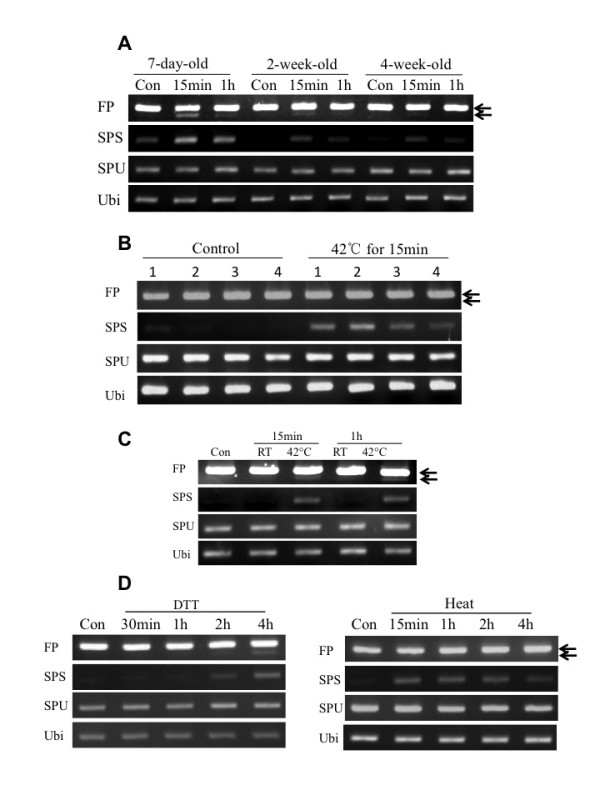
**ZmbZIP60 splicing during maize development**. (A) Maize seedlings of various ages were heat treated at 42°C for the indicated times. ZmbZIP60 mRNA splicing was analyzed as in Figure 2. (B) Following the heat stress of seedlings for 15 minutes, the splicing of ZmbZIP60 mRNA was observed in the basal (1), transitional (2), maturing (3), mature (4) regions of the third leaf [33]. (C) Leaves were detached from a seven-day-old seedling and incubated at RT or 42°C for the times indicated. (D) The tassel was detached from a mature maize plant and sprayed with DTT or subjected to heat treatment. RNA was extracted at various times after treatment and ZmbZIP60 mRNA splicing was detected using RT-PCR assays as described in Figure 2.

The stress responses examined thus far were observed in young (7-day old) seedlings. To determine whether they were dependent on developmental stage, older seedlings were heat treated and tested for ZmbZIP60 splicing. It was clear that the response was more robust in shoots of the 7-day-old seedlings compared to 2-week and 4-week old seedlings (Figure 5A). Taking advantage of the characteristics of monocot leaf growth, we also determined the efficiency of the splicing response at different stages of development within individual maize leaves. The maize leaf grows from its base towards the tip, creating a developmental gradient along the leaf axis. We subjected corn seedlings to a 15 min treatment at 42°C at the 3 leaf stage (about 10 day-old seedlings). The third leaves were excised and RNA was extracted from segments representing the basal (1), transitional (2), maturing (3) and mature (4) regions according to Li et al [33]. The youngest leaf regions, which included the basal and transitional regions, showed the most splicing activity (Figure 5B). Thus, the RNA splicing response to heat stress is more robust in younger seedlings than in older and in younger parts of the leaf than in older.

For convenience, we conducted the analysis at later developmental stages on plant parts rather than whole plants. To determine whether stress responses occur in detached organs, we excised leaves at their base, heat treated them and then extracted RNA from the apical half of the leaf. We observed that the detached leaves did, indeed, respond to the heat treatment by splicing ZmbZIP60 mRNA while the detached untreated leaves did not (Figure 5C). (In preliminary experiments, we found that there was some ZmbZIP60 mRNA splicing in tissue near the cut site at the base of the leaf, however, the background was fairly low and slow to respond.) As a representative of reproductive tissues in mature plants, we subjected detached tassels to heat treatment and found that heat could elicit a modest response within 15 min while spraying the tassel with DTT elicited a slower and equally modest response at 2-4 hrs (Figure 5D). Therefore, our experiments showed that detached organs still have the potential to respond to ER stress and can be used to detect AtbZIP60 splicing under different conditions in our RNA splicing assay.

### BIP genes

The genes encoding chaperones and other components of the protein folding machinery are part of the molecular signature of ER stress response in plants [14]. AtBIP3, one of three BIP genes in Arabidopsis, is the ER stress response gene in Arabidopsis that is most highly upregulated and dependent on AtbZIP60 for upregulation in response to ER stress [27,34]. We found 22 BIP-like genes in the maize genome, and the identity of the AtBIP3 ortholog is not obvious because the sequences of BIP genes are highly conserved and Arabidopsis BIP genes are more closely related to each other than to the maize BIP-like genes (Additional file 5).

**Figure 6:**
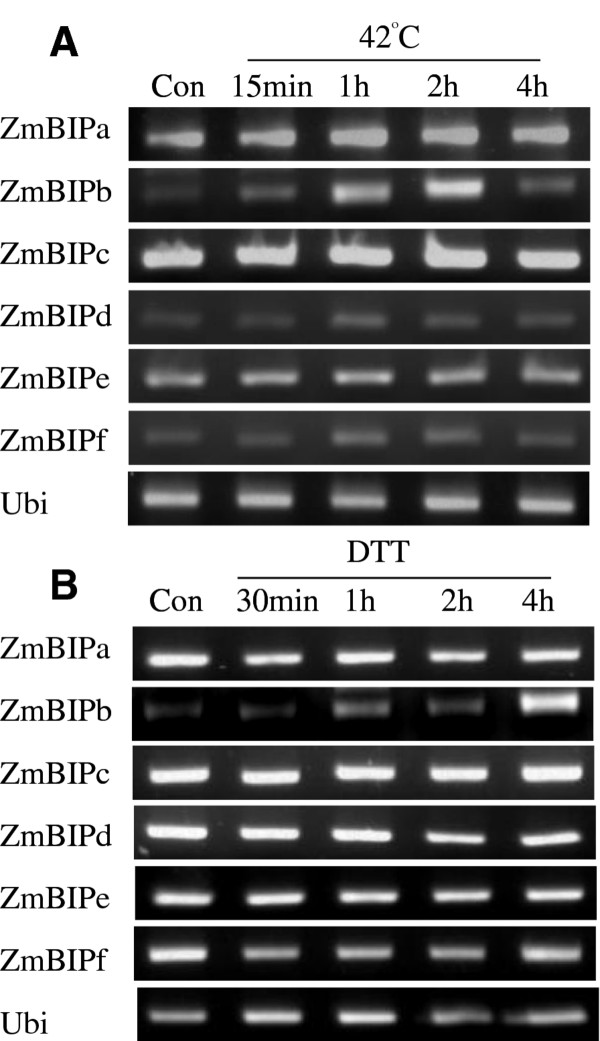
**Expression of selected BIP-like genes in maize following heat (42°C) and 2.5 mM DTT treatment for the time indicated**. RT-PCR assays were conducted using the gene-specific primers as indicated in Table S1.

To determine which, if any, maize BIP-like gene is regulated by ER stress, we picked the top six from a blast search using AtBIP3 as the query sequence and for convenience referred to these genes as ZmBIPa-f. We subjected seedlings to heat and DTT treatment. Of the six maize BIP genes, only ZmBIPb (GRMZM2G471196P01) responded significantly to ER stress. ZmBIPb was upregulated within 15 min and reached a peak at 1-2 hr after the beginning of treatment (Figure 6A). ZmBIPb was upregulated somewhat more slowly by DTT than by heat treatment reaching a peak around 4 hr (Figure 6B). It is still unclear whether ZmbZIP60 is directly involved in the upregulation of ZmBIPb by ER stress. However, the accumulation pattern of ZmBIPb mRNA is consistent with the proposition that the gene might be a ZmbZIP60 transcriptional target because the accumulation of ZmBIPb mRNA slightly lags the accumulation of spliced ZmbZIP60 mRNA. In addition, within 150 bp upstream from the start of the ZmBIPb gene in maize line B73 are five core sequences (CACG) from the p-PURE and ERSE promoter elements, which are known to regulate genes involved in ER stress responses [35].

## Discussion

In this study we show that the mRNA encoding ZmbZIP60 is spliced in response to ER stress, like its AtbZIP60 ortholog in Arabidopsis. As with AtbZIP60, a segment of ZmbZIP60 mRNA is predicted to fold into a structure that has been shown to serve as a recognition site for IRE1 in other species. The predicted RNA structure is characterized by the presence of twin "kissing" hairpin loops with three conserved bases in each loop. The RNA is cleaved in both loops, which leads to the excision of a 23b intron in Arabidopsis. A similar scenario is expected in other dicots, where orthologs display a highly conserved sequence that would lead to a similar hairpin loop structure and the excision of a 23b intron.

By contrast, the sequence and predicted structure of IRE1 recognition sites in bZIP60 orthologs in maize and other monocots are slightly different. The bridge region between the two loops is three bases shorter than in dicots leading to the removal of a 20b intron. Nonetheless, the consequence of the splicing event is similar in dicots and monocots. In both cases, the splice sites are located immediately upstream of the region encoding the TMD and excision of the introns produces a frameshift that eliminates the TMD and leads to the acquisition of a NLS. The unspliced AtbZIP60 encodes a fully functional bZIP transcription factor although it is tethered to the ER by its TMD. The functionality of the transcription factor encoded by the unspliced RNA was demonstrated by Iwata and Koizumi [36] who showed that a truncated form of bZIP60 could upregulate UPR genes when introduced into plants as a transgene.

Nagashima et al [30] pointed out that the location of the splice site and the nature of the product encoded by unspliced RNA are different in plants and animals. In Arabidopsis, and now shown here in maize, the unspliced RNA encodes a bZIP TF with a transmembrane domain. In yeast, the splice site in HAC1 is located toward the C-terminus of the protein and unspliced RNA does not encode a fully functional transcription factor with a TMD. In fact, the unspliced HAC1 RNA is poorly translated, if at all, because the intron in the unspliced RNA inhibits translation [37]. Splicing of HAC1 does not create a frameshift but rather joins a downstream, in-frame transcription activation domain to the DNA binding domain.

In mammalian cells, unspliced XBP1 mRNA, like Hac1 in yeast, does not encode a fully functional transcription factor and while the product of the unspliced RNA does not have a TMD, it does have a hydrophobic region that appears to be important for membrane binding. It has been proposed by Yanagitani et al. [38] that the nascent unspliced form of XBP1 (XBP1u) binds its own RNA and leads it to the ER membrane thereby enhancing the cytoplasmic splicing of XBP1 mRNA. In any case, splicing of XBP1 leads to a frameshift that endows the protein encoded by the spliced mRNA with an activation domain.

### Activation of ZmbZIP60

AtbZIP60 is spliced by IRE1 and Arabidopsis has two IRE1 genes, IRE1a and b [26]. Deng et al. [27] reported that most of the AtbZIP60 splicing in seedlings was mediated by IRE1b. Nagashima et al. [30] obtained somewhat different results suggesting that IRE1a and b functioned redundantly in seedlings. However, their PCR assays were nearly at full saturation for appearance of the spliced RNA, so they may have not been able to distinguish between the contribution of the IRE1a and b. Suffice it to say, that the transcription profiles for IRE1a and b are quite different in Arabidopsis. IRE1b is widely expressed, while IRE1a is more specifically expressed in flowers http://bbc.botany.utoronto.ca/efp/cgi-bin/efpWeb.cgi?dataSource=Developmental_Map. Thus, it is unlikely that IRE1a is fully functionally redundant to IRE1b in Arabidopsis seedlings. The expression patterns of the two IRE1 genes have not been reported in maize, but that information might contribute significantly to our understanding of the genes involved in this stress response in different tissues.

Treatment of maize seedlings with ER stress agents, such as TM or DTT, promotes ZmbZIP60 splicing primarily in roots. The lack of a splicing response to ER stress in shoots could result from the slow transport of ER agents from roots to shoots or from the poor uptake of the agents when topically applied by spraying onto shoots. It appears that seedling shoots are fully capable of splicing ZmbZIP60 mRNA in response to heat, therefore, it is likely that the lack of response to ER stress agents is a transport or uptake problem. However, at this point, we cannot rule out the possibility that the ER stress signals generated by stress agents might be different from those produced by heat, and roots, but not shoots can act upon those signals.

Heat is the only stress we have found that activates ZmbZIP60 splicing in testing a small panel of abiotic stresses. The response to heat stress is transient, and the amplitude (as measured by the amount of spliced RNA produced) and duration of the response are temperature dependent within a certain range. Field studies have not yet been conducted, but it would be interesting to see if the splicing response is activated by daily temperature fluctuation and/or by a period of high temperature conditions.

The ZmbZIP60 mRNA splicing response is more robust in younger seedlings than older ones and is stronger in younger parts of a leaf along a developmental gradient. The response is not entirely silenced in more mature plants because we have observed a response in tassels.

During the preparation of this paper, two other reports appeared showing IRE1-mediated splicing of OsbZIP74 (or OsbZIP50) in rice, an ortholog of ZmbZIP60 [39,40].

## Methods

### Plant materials and growth conditions

Maize inbred line B73 was used in this study. Seeds were surface-sterilized with 70% ethanol for 5 minutes, then 50% bleach water (about 3% sodium hypochlorite) for 10 minutes, and then rinsed in distilled water. The sterilized seeds were germinated, and seedlings were grown on moistened filter paper in petri dishes (15 cm × 7.5 cm) at 27°C under continuous light. For soil-grown plants, seeds were sown in SB3000 Universal Soil Mix (Sun Gro Horticulture, Bellevue, WA) watered and incubated in a growth chamber continuously at 28°C under a 16 h light/8 h dark photoperiod condition.

### Sequence analysis

The twin loop structure in ZmbZIP60 mRNA was predicted with the online algorithm Mfold http://mfold.rna.albany.edu/?q=mfold/RNA-Folding-Form using default parameters. Maize BIP genes were obtained from the maize sequence archive http://archive.maizesequence.org/index.html and identified by Blast with AtBIP1 and -3 sequences.

### Stress assays and RNA analysis

For salt and ABA treatments, roots from 7-day-old maize seedlings were immersed in 250 mM NaCl and 100 μM abscisic acid (ABA) solutions respectively. When testing for hypoxia, 7-day-old seedlings were totally submerged in water. To test the effects of dehydration, the seedlings were transferred to dry filter paper for indicated periods of time. For heat treatment, seedlings were transferred to preheated solutions and placed in an incubator at elevated temperatures as indicated.

RNA was prepared by grinding harvested tissues into powder in liquid nitrogen and RNA was extracted with Qiagen RNeasy Mini Kit according to the manufacturer's protocol. Reverse transcription reactions were performed using 2 μg total RNA with iScript cDNA Synthesis kit (Bio-rad). Primers for flanking primer assays were: FP-forward (5'-GCAGAGTGCCGTCGCCTCAGCTAC-3'), FP-reverse (5'-CCAGCCAAAGCAGGGAAACCAGC-3'). Primers for the specific unspliced RNA assay were: SPU-reverse (5'-GGCAGGGTTTCCGTGAGTAC-3') and the FP-forward primer from above. Primers for the specific spliced RNA assay were: SPS-reverse (5'-GCAGGGAAACCAGCGGCTGAC-3' and the FP-forward primer from above. (To distinguish between the spliced and unspliced form of ZmbZIP60, a mismatch was introduced at the fourth nucleotide of the 3' end of the primer). Primers for amplifying maize ubiquitin and BIP homologs were listed in Additional file 6.

## Competing interests

The authors declare that they have no competing interests.

## Authors' contributions

YL carried out the experimental work and participated in the design of the study, SH identified the secondary structure in ZmbZIP60 mRNA, SHH conceived the study and drafted the manuscript. All authors read and approved the final manuscript.

## Authors' information

S Humbert's current address: Pioneer Hi-Bred International, Johnston, IA 50131, USA

## Supplementary Material

Additional file 1 **Phylogenetic analysis of bzip60 orthologs in monocots and dicots**. Branched dendrogram was produced using Clustal W. Sequences were obtained from GenBank by conducting BLAST search with AtbZIP60. Click here for file

Additional file 2 **Phylogenetic analysis of IRE1 orthologs in monocots and dicots**. Branched dendrogram was produced using Clustal W. Sequences were obtained from GenBank by conducting BLAST search with AtbIRE1b. Click here for file

Additional file 3 **Sequences of IRE1 recognition sites in bzip60-related genes in dicots and monocots**. Predicted loops in the secondary structures of the RNAs are indicated in Figure 1. Click here for file

Additional file 4 **Tests for Zmbzip60 splicing following treatments by (A) hypoxia (water immersion) and cold (4°C) for various times or following treatments with (B, C), high saline (250 mM NaCl), dehydration (dry filter paper) or 100 μM abscisic acid (ABA)**. Click here for file

Additional file 5 **Phylogenetic analysis of BIP-like genes in maize**. Rooted phylogenetic tree was produced using Unweighted Pair Group Method with Arithmetic Mean in ClustalW http://www.genome.jp/tools/clustalw/. Sequences were obtained from the maize sequence archive by conducting BLAST search with AtBIP1 and -3. Genes are identified by the accession numbers in the sequence archive database. The six ZmBIP-like genes that top the BLAST list are labeled ZmBIPa-f. ZmBIPb is upregulated under ER stress conditions in maize seedlings. ZmBIPa = GRMZM2G087891_P01; ZmBIPb = GRMZM2G471196_P01; ZmBIPc = GRMZM2G018490_P01; ZmBIPd = GRMZM2G310431_P01; ZmBIPe = GRMZM2G056039_P01; ZmBIPf = GRMZM2G366532_P01; AtBIP1 = AT5G28540.1; AtBIP2 = AT5G42020.1; AtBIP3 = AT1G09080.1. Click here for file

Additional file 6 **Other PCR primers used in this study**. Click here for file
