# Supplementary figures and images for: ZmbZIP60 mRNA is spliced in maize in response to ER stress
Source: BMC Res Notes. 2012 Mar 14;5:144. doi: 10.1186/1756-0500-5-144 (PMC3369818; doi:10.1186/1756-0500-5-144)

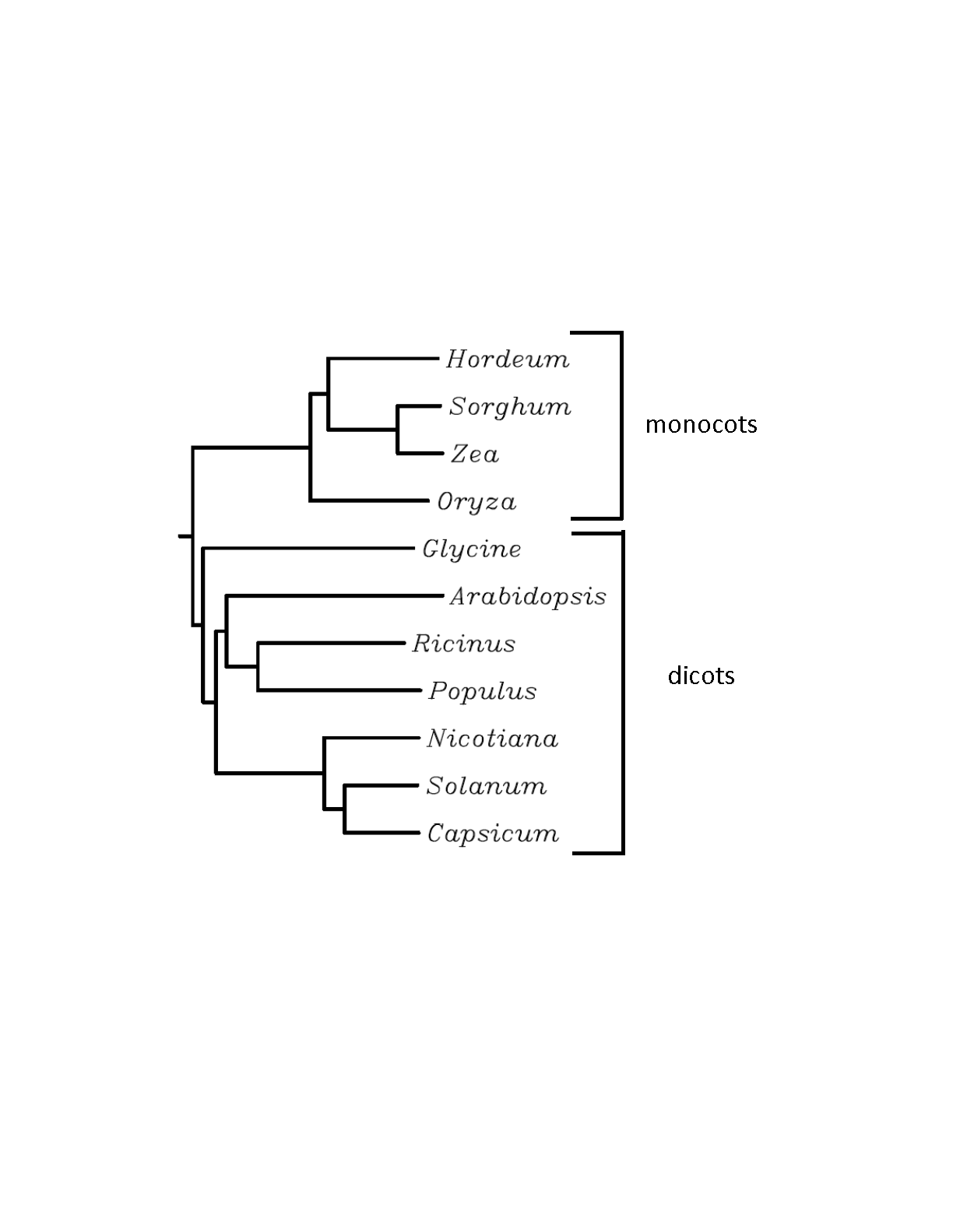

Supplement: Additional file 1 — Phylogenetic analysis of bzip60 orthologs in monocots and dicots. Branched dendrogram was produced using Clustal W. Sequences were obtained from GenBank by conducting BLAST search with AtbZIP60. [file 1756-0500-5-144-S1.PNG]

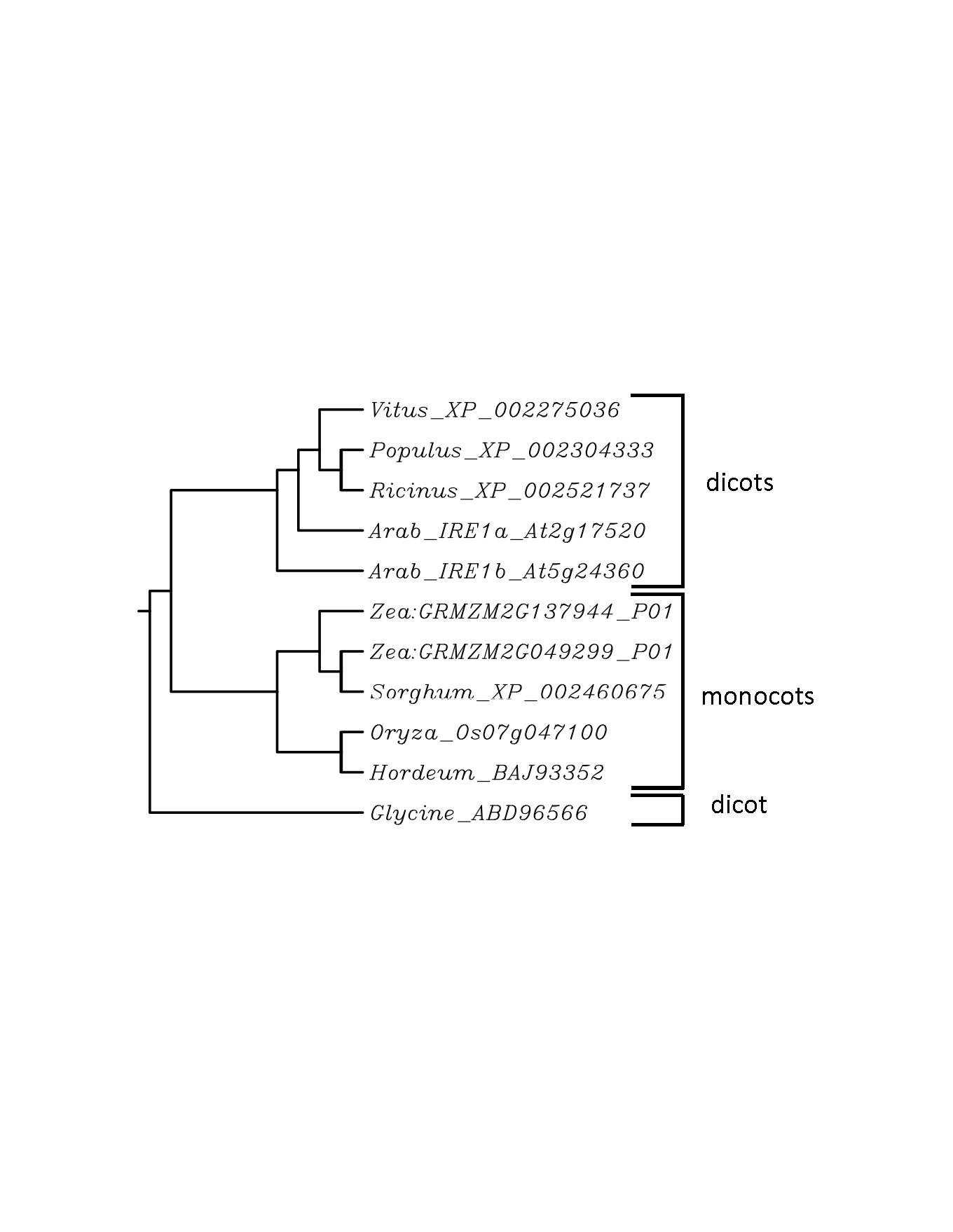

Supplement: Additional file 2 — Phylogenetic analysis of IRE1 orthologs in monocots and dicots. Branched dendrogram was produced using Clustal W. Sequences were obtained from GenBank by conducting BLAST search with AtbIRE1b. [file 1756-0500-5-144-S2.PNG]

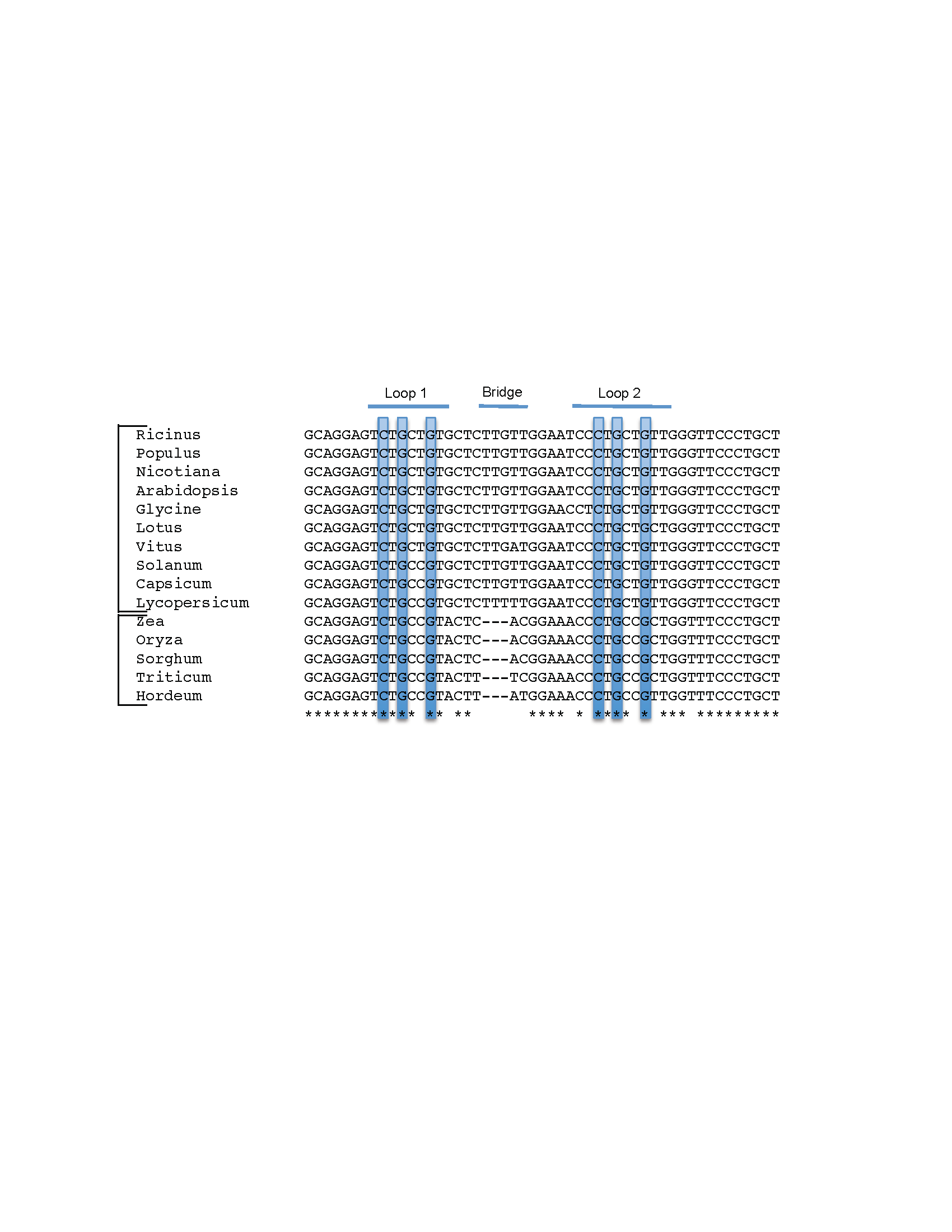

Supplement: Additional file 3 — Sequences of IRE1 recognition sites in bzip60-related genes in dicots and monocots. Predicted loops in the secondary structures of the RNAs are indicated in Figure 1. [file 1756-0500-5-144-S3.PNG]

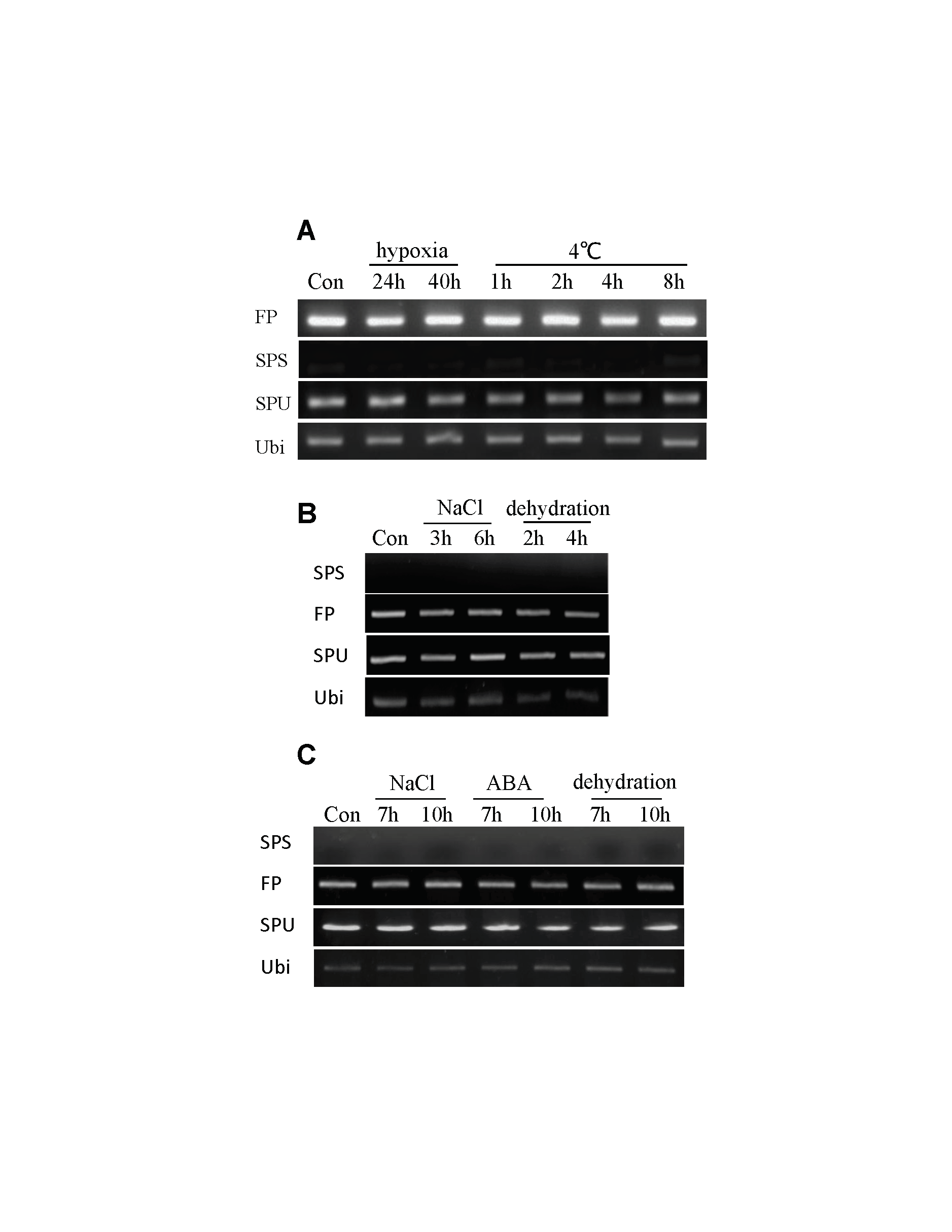

Supplement: Additional file 4 — Tests for Zmbzip60 splicing following treatments by (A) hypoxia (water immersion) and cold (4°C) for various times or following treatments with (B, C), high saline (250 mM NaCl), dehydration (dry filter paper) or 100 μM abscisic acid (ABA). [file 1756-0500-5-144-S4.PNG]

## Slide 1
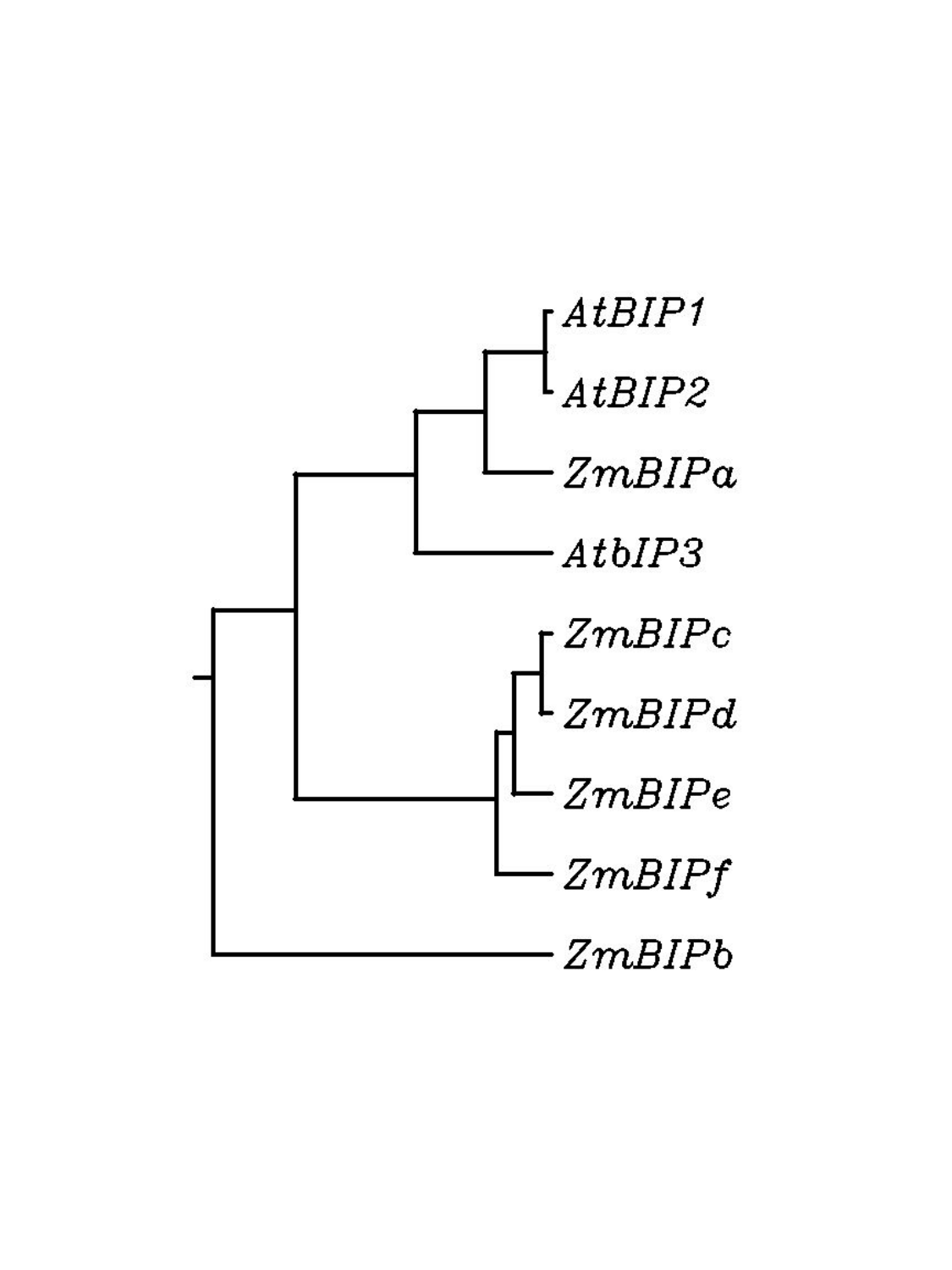

Supplement: Additional file 5 — Phylogenetic analysis of BIP-like genes in maize. Rooted phylogenetic tree was produced using Unweighted Pair Group Method with Arithmetic Mean in ClustalW http://www.genome.jp/tools/clustalw/. Sequences were obtained from the maize sequence archive by conducting BLAST search with AtBIP1 and -3. Genes are identified by the accession numbers in the sequence archive database. The six ZmBIP-like genes that top the BLAST list are labeled ZmBIPa-f. ZmBIPb is upregulated under ER stress conditions in maize seedlings. ZmBIPa = GRMZM2G087891_P01; ZmBIPb = GRMZM2G471196_P01; ZmBIPc = GRMZM2G018490_P01; ZmBIPd = GRMZM2G310431_P01; ZmBIPe = GRMZM2G056039_P01; ZmBIPf = GRMZM2G366532_P01; AtBIP1 = AT5G28540.1; AtBIP2 = AT5G42020.1; AtBIP3 = AT1G09080.1. [file 1756-0500-5-144-S5.PPT]

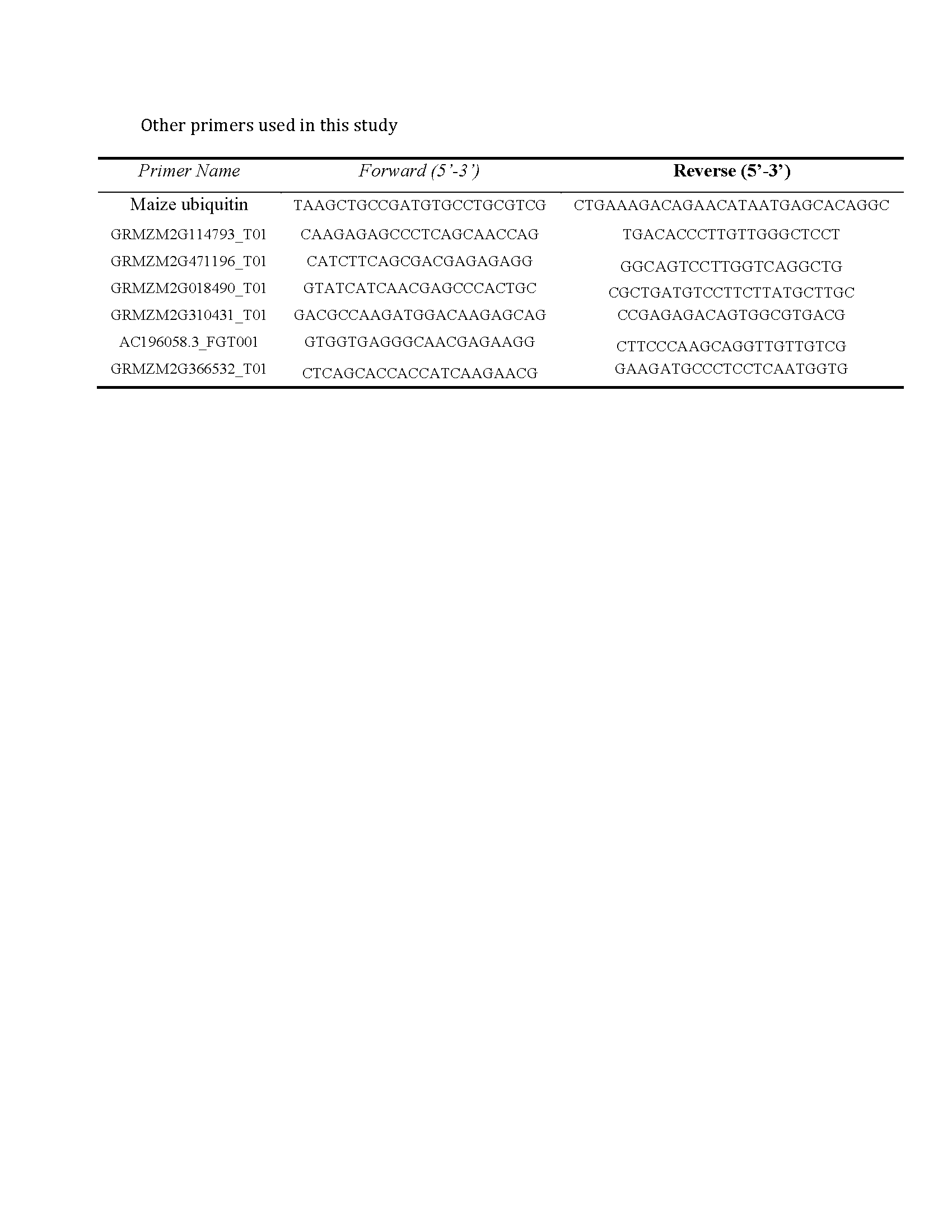

Supplement: Additional file 6 — Other PCR primers used in this study. [file 1756-0500-5-144-S6.PNG]
